# The underlying regulatory mechanisms of colorectal carcinoma by combining Vitexin and Aspirin: based on systems biology, molecular docking, molecular dynamics simulation, and *in vitro* study

**DOI:** 10.3389/fendo.2023.1147132

**Published:** 2023-07-26

**Authors:** Dengsheng Chen, Ying Chen, Fang Huang, Xiaoling Zhang, Yulv Zhou, Luning Xu

**Affiliations:** ^1^ Department of Clinical Pharmacy, Sanming First Hospital, Affiliated Hospital of Fujian Medical University, Sanming, Fujian, China; ^2^ Department of Chinese Medicine and Anorectology, Sanming First Hospital, Affiliated Hospital of Fujian Medical University, Sanming, Fujian, China

**Keywords:** colorectal cancer, vitexin, Aspirin, network pharmacology, molecular docking, experimental verification

## Abstract

**Introduction:**

Colorectal cancer (CRC) is a highly prevalent digestive system malignancy. Aspirin is currently one of the most promising chemopreventive agents for CRC, and the combination of aspirin and natural compounds helps to enhance the anticancer activity of aspirin. Natural flavonoids like vitexin have an anticancer activity focusing on colorectal carcinoma.

**Methods:**

This study investigated the potential mechanism of action of the novel combination of vitexin and aspirin against colorectal cancer through network pharmacology, molecular docking, molecular dynamics simulation, and *in vitro* experiments.

**Results:**

The results of network pharmacology suggested that vitexin and aspirin regulate multiple signaling pathways through various target proteins such as NFKB1, PTGS2 (COX-2), MAPK1, MAPK3, and TP53. Cellular experiments revealed that the combined effect of vitexin and aspirin significantly inhibited HT-29 cell growth. Vitexin dose-dependently inhibited COX-2 expression in cells and enhanced the down-regulation of COX-2 and NF-κB expression in colorectal cancer cells by aspirin.

**Discussion:**

This study provides a pharmacodynamic material and theoretical basis for applying agents against colorectal cancer to delay the development of drug resistance and improve the prognosis of cancer patients.

## 1 Introduction

The most typical malignant tumor of the digestive system, colorectal cancer (CRC), is the third most common cause of cancer-related death. Only 63.5% of patients survived for five years; age was inversely connected with survival (1, 2). The latest report by the World Health Organization (WHO) shows that the overall incidence and mortality rate of CRC has increased to third and second place, respectively. By 2020, CRC will account for 10% of new cancer cases and 9.4% of deaths worldwide (3). The incidence and mortality of colorectal cancer in China are rising (4). Common colorectal cancer treatments include surgery, radiotherapy and chemotherapy, targeted therapy, immunotherapy, and Chinese medicine. Among them, surgical resection of primary tumors and oligo nuclear metastases treatment are the first choices for CRC patients (5). Studies have shown that the efficacy of chemotherapy in colorectal cancer patients varies with cancer subtypes and that cytotoxicity, drug resistance, and adverse effects are the main issues affecting the effectiveness of chemotherapy (6, 7).

Aspirin has been reported as a potential chemo preventive agent for several cancers, including colorectal cancer (8), non-small cell lung cancer (9), gastric cancer (10), breast cancer (11), and hepatocellular carcinoma (12). Aspirin is one of the most promising chemo preventive agents for CRC, not only for primary prevention of CRC but also for reducing the risk of recurrence and metastasis after radical surgery in early-stage CRC patients (13). Studies have shown that the combination of aspirin and natural compounds helps to enhance the efficacy of aspirin (14), and the combination with other chemotherapeutic agents enhances anticancer activity (9). Recent studies have shown significant synergistic effects of flavonoids with each other and with other anticancer drugs in preventing cancer development, tumor progression and promoting apoptosis. In colorectal cancer, they may reduce the incidence and risk of recurrence of colorectal cancer and may be used to enhance the current treatment of this disease (15).

Vitexin is a natural *phytoflavonoid* widely found in many edible and medicinal plants (16). Vitexin has a variety of biological activities, including antioxidant, anti-inflammatory, neuronal protection, and cardio protection, as well as fat reduction, glucose metabolism, and hepatoprotective effects (17). Studies have shown that vitexin has anti-proliferative effects on a variety of tumor cells (18), such as glioma (19), liver cancer (20), nasopharyngeal carcinoma (21), and colon cancer, with a wide range of tumor therapeutic effects. More importantly, several studies have pointed out that vitexin can prevent colitis-associated carcinogenesis in mice by regulating macrophage polarization (22) and effectively inhibit the proliferation of colorectal cancer *in vivo* or *in vitro* (23, 24). Therefore, it is hypothesized that vitexin is a potential anticancer chemotherapeutic agent, including colorectal cancer, and that its novel combination with aspirin enhances the anticancer activity with a focus on the colorectal carcinoma effect of aspirin.

Network pharmacology describes the complex interactions between drugs, organisms, and diseases from a network perspective, helping to provide a more comprehensive understanding of the pathological basis of diseases and the effects of drug therapy. Network pharmacology, which studies the pharmacodynamic mechanisms of action of active compounds by constructing and resolving drug-target-disease biological network enrichment relationships, has been applied to study the synergistic and complementary nature of traditional Chinese medicine (TCM) and the relationship between multiple targets and multiple mechanisms of action in TCM (25, 26). Molecular docking is another new approach used in TCM research to predict ligand-target interactions at the molecular level and is widely used in drug discovery to identify therapeutically significant letter compounds (27). It is a promising research direction from the perspective of network pharmacology and molecular docking. However, validation of the findings in cellular or animal experiments is required due to the virtual nature of network pharmacology and molecular docking.

Due to the anticancer activity with a focus on colorectal carcinoma of vitexin and aspirin, we proposed whether the novel combination of vitexin and aspirin could enhance the anticancer activity with a focus on colorectal carcinoma effect. This study explored the underlying mechanism of action of vitexin and aspirin against colorectal cancer through network pharmacology, molecular docking, and molecular dynamics simulation. To provide a theoretical foundation for improving the antitumor effect of aspirin and lowering the side effects like bleeding caused by aspirin, we then investigated the effect of vitexin on the biological behavior of colorectal cancer cell line HT-29 cells as well as its effects on protein and mRNA of colorectal cancer cells after its collective action with aspirin.

## 2 Results

### 2.1 Network pharmacology analysis

#### 2.1.1 Acquisition of vitexin, aspirin, and disease targets and construction of “traditional Chinese medicine - component - target - disease” network diagram

**Figure 1:**
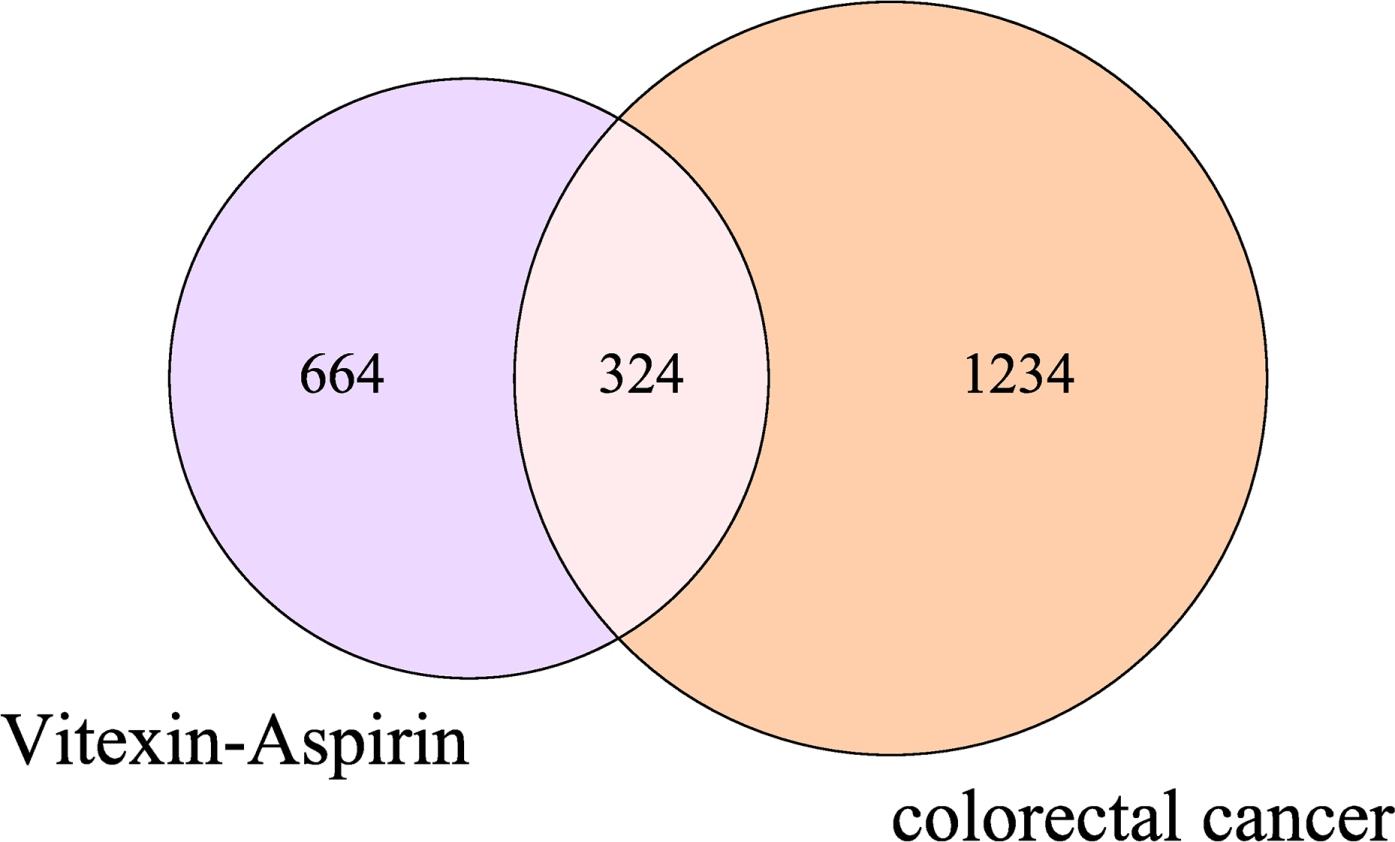
Venn diagram of vitexin, aspirin, and colorectal cancer targets. The purple and pink areas in the Figure represent target genes of vitexin and aspirin; the pink and orange areas represent target genes related to colorectal cancer; the pink areas represent duplicate target genes of vitexin and aspirin with colorectal cancer.

**Figure 2:**
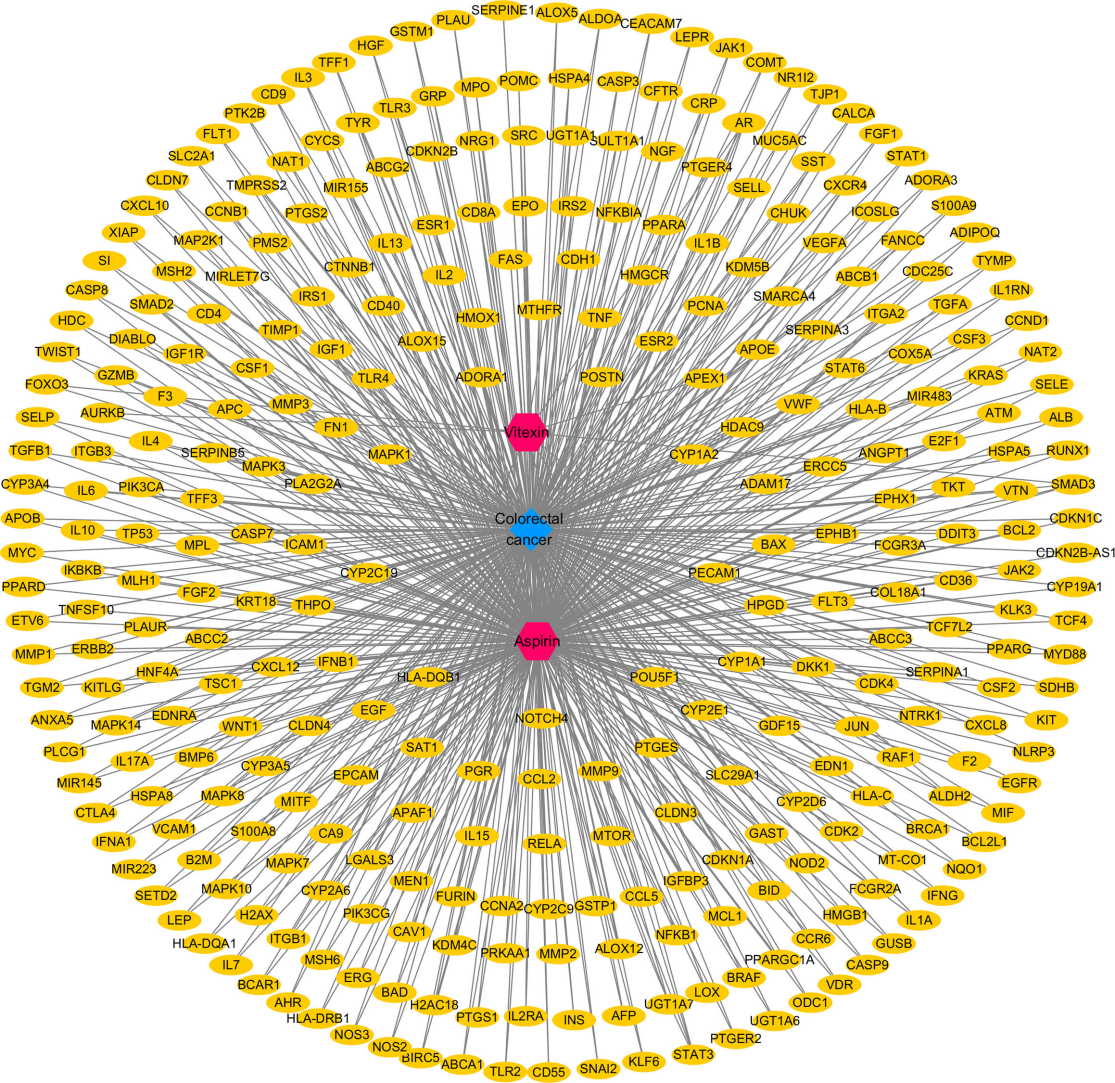
Vitexin- aspirin - colorectal cancer - target network.

By searching the database and merging and removing duplicate values, 17 targets of vitexin and 978 targets of aspirin were obtained. 1,494 colorectal cancer-related targets (median value of Relevance score ≥ 10.00426) were retrieved from the Gene Cards database, and 158 related targets (targets marked with an “*”) were retrieved from the OMIM database, and the two were merged, and duplicate values were removed to obtain a total of 1558 colorectal cancer-related targets. The vitexin targets and aspirin targets were combined and imported into the bioinformatics website (http://www.bioinformatics.com.cn/?p=2) with the obtained colorectal cancer targets for an intersection to construct a Venn diagram, and 324 common targets were obtained, as shown in **Figure 1**. The drug-disease-target network was constructed using Cytoscape 3.2.1 software, as shown in **Figure 2**. The network contains 327 nodes and 656 edges. These cross-targets were potential candidate targets for vitexin-enhanced aspirin against colorectal cancer.

#### 2.1.2 Gene ontology (GO) enrichment analysis

**Figure 3:**
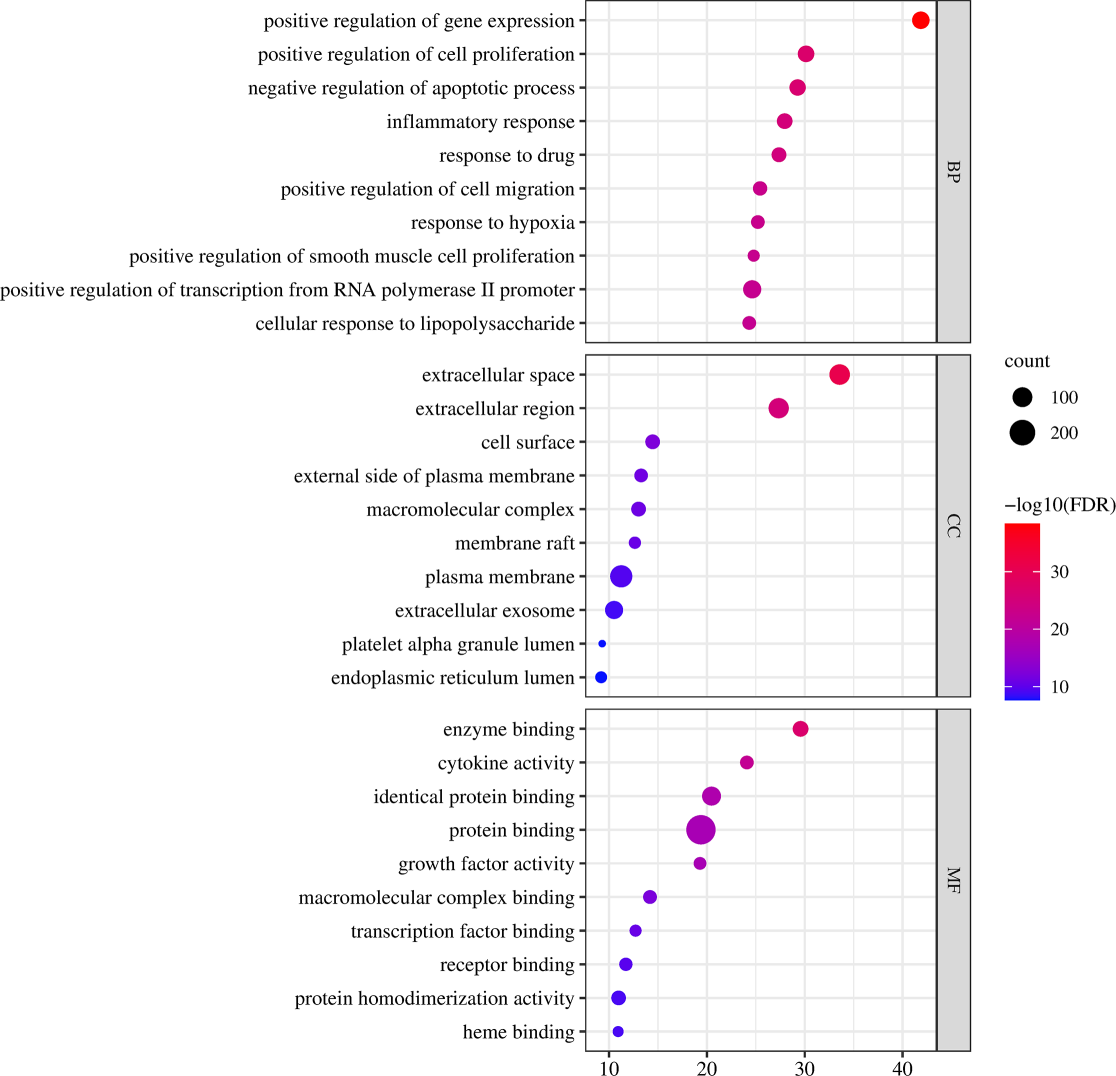
Vitexin-aspirin-colorectal cancer GO enrichment analysis.

GO functional enrichment analysis includes three components: biological process (BP), molecular function (MF), and cellular component (CC). The 324 intersecting targets of vitexin, aspirin, and colorectal cancer were imported into the DAVID database, and the GO functional enrichment analysis was performed by selecting the Functional Annotation tool, with species limited to “Homo Sapiens” and the rest set to default. 1181 BPs, 118 CCs, and 204 MFs were obtained, and the top 10 were selected for analysis, as shown in **Figure 3**. The biological process involves positive regulation of gene expression, positive regulation of cell proliferation, and negative regulation of the apoptotic process, the molecular function involves enzyme binding, cytokine activity, and identical protein binding, and the cell component involves extracellular space, extracellular region, and cell surface.

#### 2.1.3 Kyoto encyclopedia of genes and genomes pathway (KEGG) analysis

**Figure 4:**
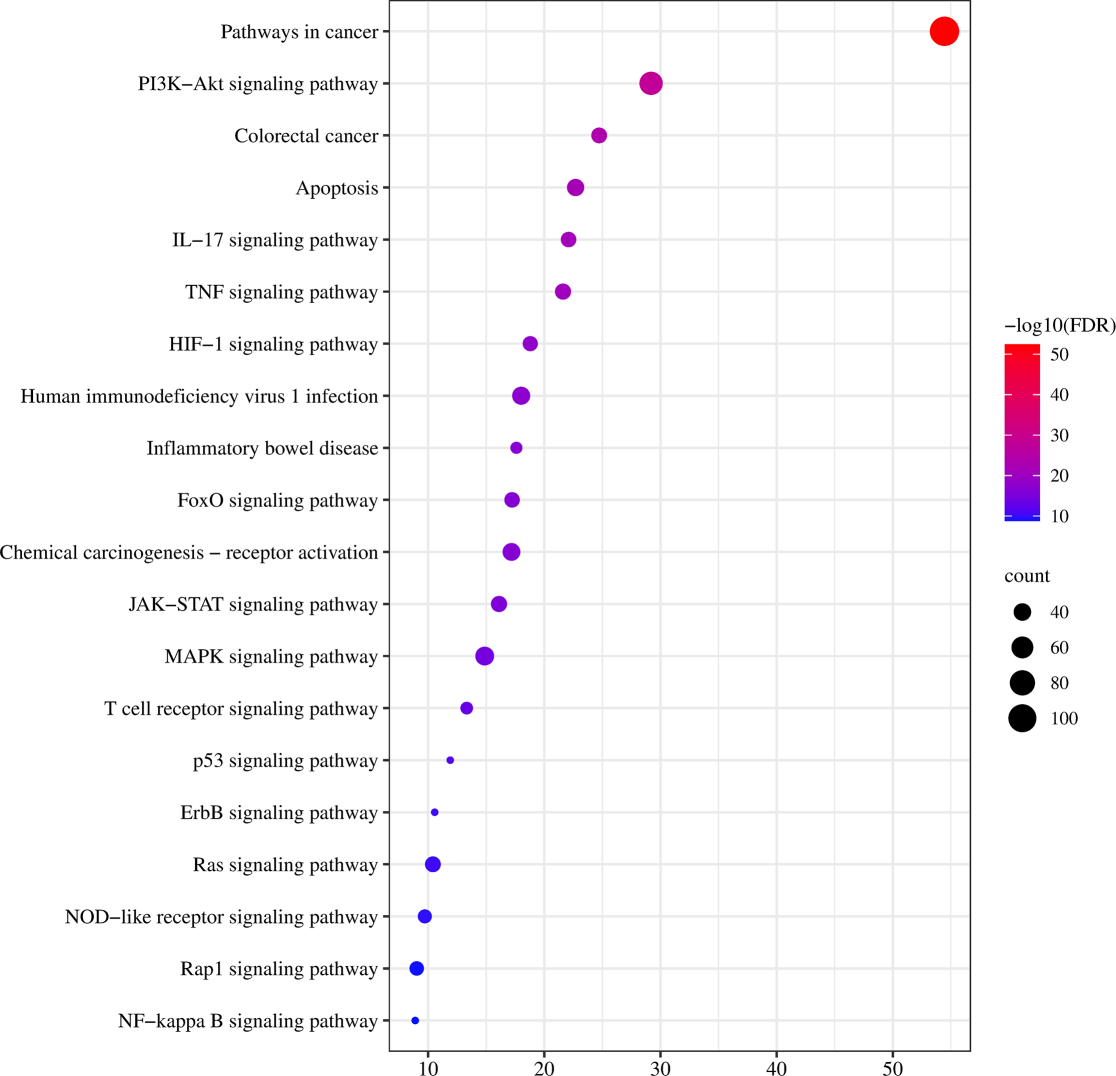
Vitexin-aspirin-colorectal cancer KEGG enrichment analysis.

**Figure 5:**
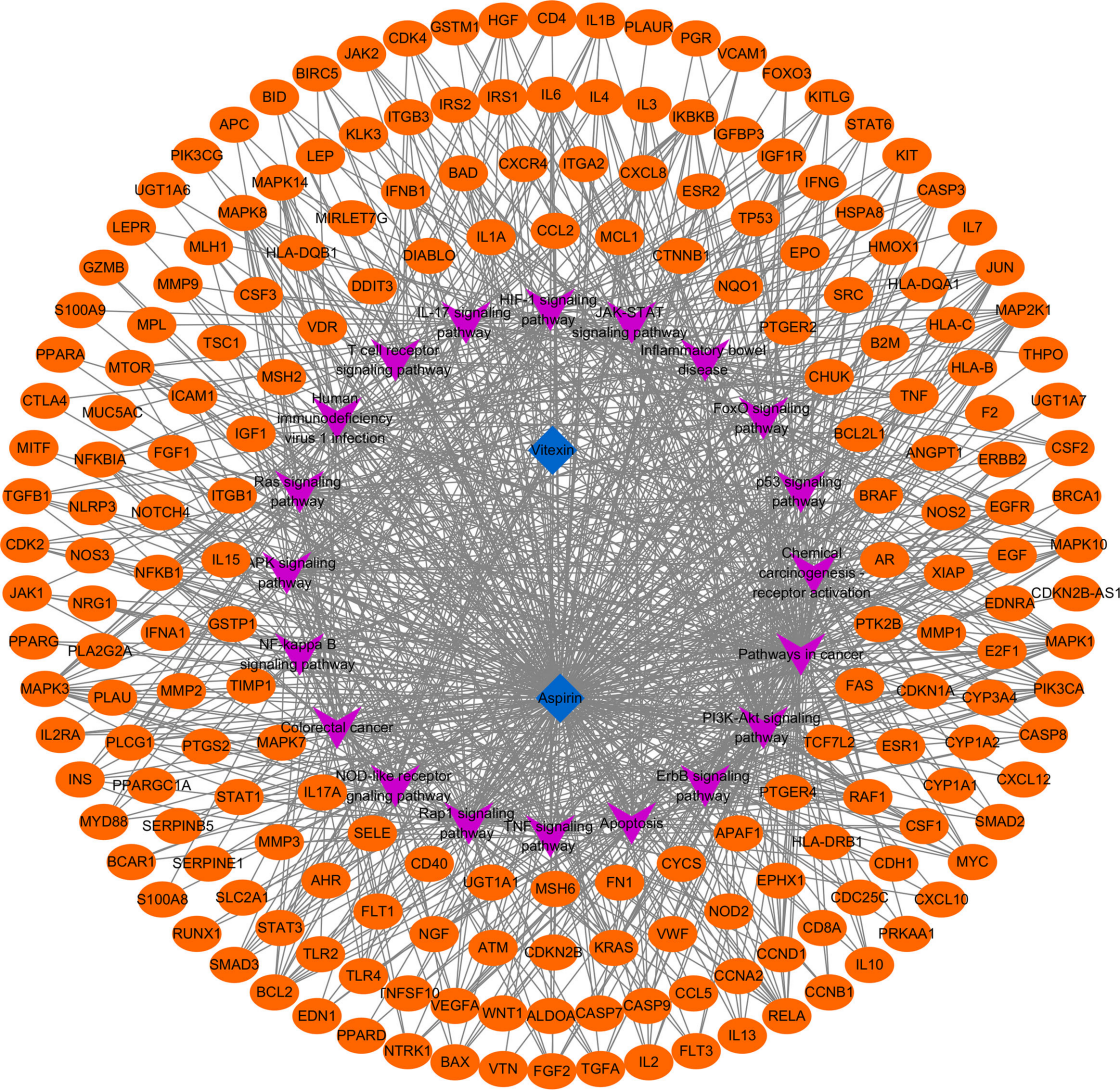
Target pathway network of vitexin to enhance the anti-colorectal cancer effect of aspirin.

To further clarify the possible pathways of vitexin-enhanced aspirin against colorectal cancer, KEGG enrichment analysis was performed on 324 target genes using the DAVID 6.8 database, totaling 178 with *P*-values less than 0.05. The top 20 were selected for visual analysis by sorting according to FDR values and removing irrelevant pathways, as shown in **Figure 4**. Key pathways involved in Pathways in cancer, PI3K-Akt signaling pathway, Colorectal cancer, IL17 signaling pathway, TNF signaling pathway, and NF-κB signaling pathway. We further analyzed the genes involved in these pathways, including NFKB1, PTGS2, MAPK1, MAPK3, and TP53 (**Figure 5**), suggesting that PTGS2, NFKB1, MAPK1, MAPK3, and TP53 may be the key target genes for the new combination of vitexin and aspirin to enhance the efficacy of colorectal cancer.

### 2.2 Molecular docking

**Figure 6:**
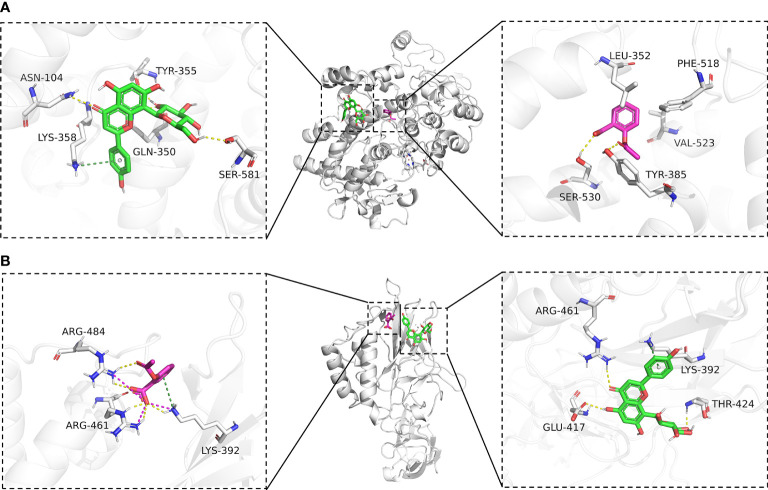
Molecular docking diagram. **(A)** Binding mode of Aspirin-Vitexin-COX-2; **(B)** Binding mode of Aspirin-Vitexin- NFKB1. The middle Figure is the overall view; the left Figure shows the details of the interaction between vitexin and protein; the right Figure shows the details of the interaction between Aspirin and protein; the green stick in the Figure is a small molecule of Vitexin; the magenta stick is small molecule Aspirin; the white cartoon is protein, the yellow line indicates hydrogen bonding interaction, the magenta dashed line indicates salt-bridge interaction and a dark green dashed line indicates cation-pi interaction.

To further explore the affinity of vitexin and aspirin to the core targets PTGS2 (COX-2) and NFKB1, we docked PTGS2 (COX-2) and NFKB1 with vitexin and aspirin simultaneously. Vina 1.1.2 software was used to predict the Aspirin-Vitexin-COX-2 and Aspirin-Vitexin-NF-κB1 ternary complexes (**Figure 6**).

In the Aspirin-Vitexin-COX-2 and Aspirin-Vitexin- NFKB1 ternary complexes, the binding affinity scores of aspirin and vitexin to COX-2 were -6.8 and -7.5 kcal/mol, respectively, and the binding affinity scores of aspirin and vitexin to COX-2 were -4.9 and -6.0 kcal/mol, which implied that aspirin and vitexin were able to bind to both COX-2 and NFKB1 proteins. **Figure 6A** show the Aspirin -Vitexin-COX-2 ternary complex interaction diagram. Aspirin (magenta) binds among the active sites of COX-2, consistent with the previously reported aspirin binding site (28). We can observe that aspirin forms hydrogen bonding interactions with TYR-385 and SER-530. In addition, the benzene ring of aspirin is in the hydrophobic pocket formed by LEU-352, PHE-518, and VAL-523. This interaction contributes to the acetylation modification of SER-530 by aspirin. In contrast to aspirin binding in the internal pocket of COX-2 protein, vitexin binds in the COX-2 protein surface cavity, the surface of the channel through which aspirin enters the pocket. The left panel show that vitexin forms hydrogen bonding interactions with ASN-104, TYR-355, GLN-350, and SER-581 on the COX-2 protein and also cation-pi interactions with Lys-358. These interactions allow vitexin to bind stably to the surface of the COX-2 protein channel and facilitate aspirin not missing the target. The combined action of the two ligands facilitates preventing COX2 substrate arachidonic acid from entering the protein to produce its effect. In the interaction diagram of the Aspirin-Vitexin-NFKB1 ternary complex, the adjacency of aspirin and vitexin molecules can be seen adjacent to each other in the left panel of **Figure 6B**. Aspirin forms hydrogen bonding interactions with LYS-392, ARG-461, and ARG-484 on the NFKB1 protein and interacts with ARG-484 and LYS-394 cation-pi interaction. From the right panel of **Figure 6B**, we can see that vitexin forms hydrogen bonding with GLU-417, ARG-461, and THR-424 on NFKB1 protein and cation-pi interaction with LYS-392. We speculate that aspirin and vitexin molecules are adjacent to each other and simultaneously interact with the protein surface in numerous ways, which may possess synergistic inhibition of protein conformation and thus prevent its biological effects.

### 2.3 Molecular dynamics simulation (MD)

To further elaborate on the effect of the bimolecule molecule on the protein, molecular dynamics simulations were used to show the difference in stability of the two proteins under the action of aspirin and under the simultaneous action of Aspirin/Vitexin, as well as the binding energy.

**Figure 7:**
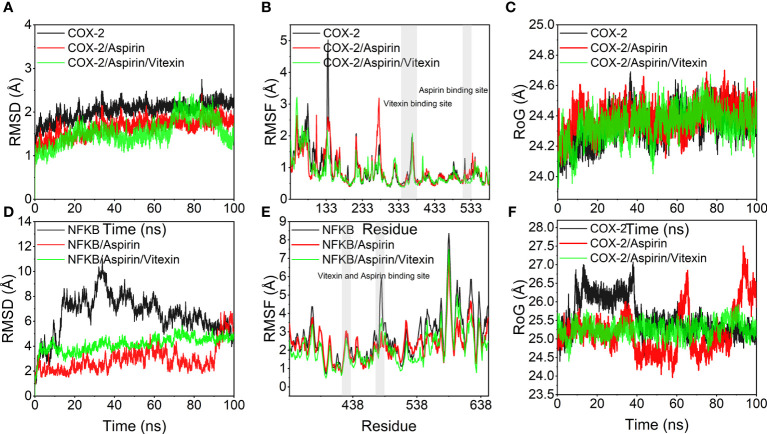
Molecular dynamics simulation results. **(A, D)** variation of root mean square deviation (RMSD) of the simulated system with time during molecular dynamics simulation; **(B, E)** root mean square fluctuation (RMSF) of the protein system during molecular dynamics simulation, the gray background indicates the small molecule ligand binding region; **(C, D)** variation of the radius of gyration (RoG) of the system with time during molecular dynamics simulation.

The root means square deviation of molecular dynamics simulations can reflect the simulated system’s motion degree, with larger RMSD indicating greater structural changes, larger fluctuations, and more violent fluctuations indicating violent motion and, conversely, smooth motion. **Figures 7A, D** show the variation of RMSD with time during the simulation for COX-2, COX-2/Aspirin, COX-2/Aspirin/Vitexin, NFKB1, NFKB1/Aspirin, and NFKB1/Aspirin/Vitexin systems. It can be seen from the diagram that COX-2/Aspirin, the RMSD values of COX-2/Aspirin/Vitexin were low and fluctuated steadily, implying that the system was stable, and the RMSD of the system after aspirin and vitexin showed a smaller trend, indicating that the complex COX-2/Aspirin/Vitexin binding was stable; The RMSD values of NFKB1, NFKB1/Aspirin and NFKB1/Aspirin/Vitexin were lower than those of NFKB1. Among them, the RMSD fluctuation of the NFKB1 system decreased significantly after binding two small ligands simultaneously, which means that aspirin and vitexin can cooperate to make NFKB1 protein more stable. From **Figures 7B, E**, we can find that the overall RMSF of the protein decreases after binding small molecules, and it is noteworthy that the small molecule binding region also shows a trend of decreasing RMSF. It means that the amino acid flexibility of several protein regions becomes smaller under the binding of small molecules, especially the joint action of two small molecules, in which the RMSF of NFKB1 protein is most affected by the binding of two molecules. This significant decrease in RMSF inhibits protein structural changes and thus protein function. In addition, we also calculated the RoG (radius of gyration) of each system over time. We can see from **Figure 7C** that the RoG of COX-2, COX-2/Aspirin, and COX-2/Aspirin/Vitexin are less different, and small molecule has little effect on the tightness of COX-2. Combined with the analysis of the binding mode of small molecule aspirin and COX-2, we judged that aspirin inhibits COX-2 protein mainly by binding to the active site of the protein and thus blocking the entry of the substrate. Under the simultaneous combination of vitexin and aspirin, the RMSD mentioned above, and RMSF were smaller, indicating that the inhibitory effect was more obvious. For NFKB1, NFKB1/Aspirin, and NFKB1/Aspirin/Vitexin, the RoG fluctuation of NFKB1/Aspirin/Vitexin was the smallest (**Figure 7F**), again implying a high degree of NFKB1/Aspirin/Vitexin tightness and that the binding of aspirin and vitexin bimolecules molecules could stabilize the conformation of NFKB1 protein by synergistic action and thus inhibit the protein action.

### 2.4 *In vitro* experimental validation

#### 2.4.1 Enhanced inhibition of colorectal cancer cell proliferation by aspirin with vitexin

**Figure 8:**
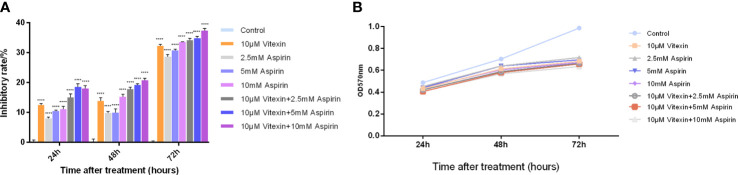
Enhanced inhibition of colorectal cancer cell proliferation by aspirin with vitexin. **(A)** Histogram of the inhibition of colorectal HT-29 and HCT-116 cell proliferation by vitexin and different concentrations of aspirin; **(B)** Line graph of the inhibition of colorectal HT-29 and HCT-116 cell proliferation by vitexin and different concentrations of aspirin. Each bar represents the mean ± SD from three independent assays. ****P < 0:001.

HT-29 colorectal cancer cell line was selected and divided into four groups: negative control group (The same amount of DMSO as the drug group), vitexin (10 μM) group, aspirin (2.5,5, 10 mM) group, and combination group (vitexin 10 μM + aspirin (2.5,5, 10 mM)). The effects of vitexin and aspirin on the proliferation of HT-29 colorectal cancer were examined by the MTT method. The results showed that the effects of different treatments on the proliferation of HT-29 cells expressing COX-2 were examined at 24, 48, and 72 h of treatment, respectively. The results showed (**Figure 8**) that the inhibition rate of the value-added HT-29 cells by vitexin applied alone ranged from 11.85-33.31%, and its inhibitory effect was enhanced with the increase of incubation time, and the highest proliferation inhibition rate reached 33.31% for the treatment of HT-29 cells for 72 h. The inhibition rate of proliferation of the two cell types by different concentrations of aspirin alone ranged from 7.29-33.73%, and the strongest inhibitory effect on the proliferation of HT-29 cells was achieved by incubation for 72 h after treatment, reaching 33.73%. The combined application inhibited the proliferation of HT-29 colorectal cancer cells by 13.67-38.51%, and the strongest inhibitory effect on HT-29 cells was achieved by incubating the cells for 72 h after treatment, reaching 38.51%. The results showed that the combination of vitexin and aspirin had a certain inhibitory effect on the proliferation of colorectal cancer cells after 48 and 72 h of treatment, suggesting that vitexin can enhance the inhibitory effect of aspirin on the proliferation of colorectal cancer cells and there is a synergistic enhancement with the inhibitory effect of aspirin on the proliferation of colorectal cancer cells.

#### 2.4.2 Inhibition of COX-2 expression in colorectal cancer HT-29 cells by vitexin

**Figure 9:**
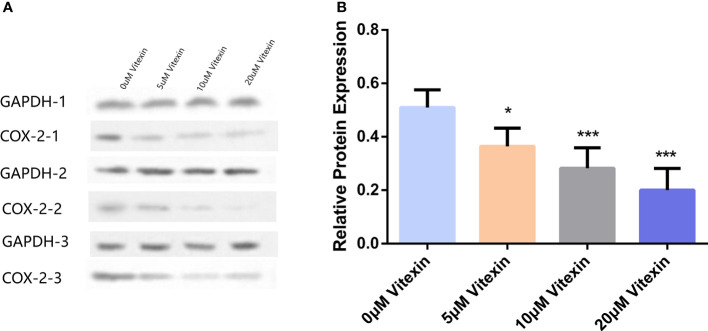
Inhibition of COX-2 expression in colorectal cancer HT-29 cells by vitexin. **(A)** Electrophoresis of COX-2 protein in HT-29 cells, GAPDH was used as a loading control. **(B)** Quantification of the corresponding grayscale values of the protein blots. Western blots were performed in triplicate. Each bar represents the mean ± SD from three independent assays. *P < 0:05 ; ***P < 0:001.

Colorectal cancer HT-29 cells with high COX-2 expression were selected to detect the inhibition of COX-2 expression by vitexin. The HT-29 cells were grown in 6-well plates and treated with different concentrations of vitexin, and the expression of COX-2 was detected by Western blot, and the results showed (**Figure 9**) that vitexin could dose-dependently inhibit the expression of COX-2 in the HT-29 cell line.

#### 2.4.3 Enhanced inhibition of COX-2 expression by aspirin with vitexin

**Figure 10:**
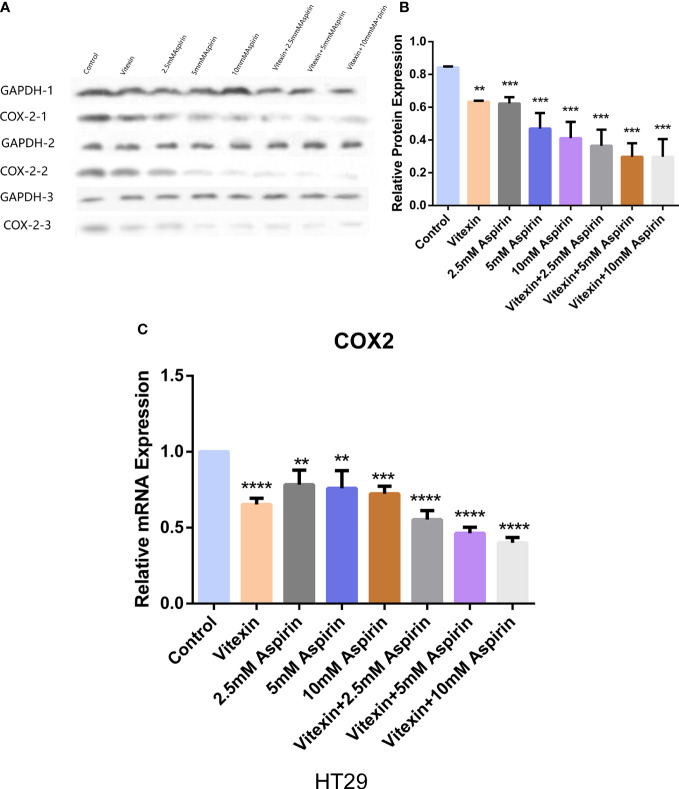
Western blot and RT-qPCR method to detect the effect of vitexin and aspirin on COX-2 in colorectal cancer HT-29 cells. **(A, B)** Western blot assay to detect the effect of vitexin and aspirin on COX-2 in colorectal cancer HT-29 cells; C: RT-qPCR assay to detect the effect of vitexin and aspirin on COX-2 in colorectal cancer HT-29 cells. Each bar represents the mean ± SD from three independent assays. **P < 0:01; ***P < 0:001; ****P < 0:0001.

To detect the effect of vitexin and aspirin on COX-2 expression in HT-29 cells. The cells were grown in 6-well plates, and HT-29 cells were treated with the four treatments mentioned above, and the expression of COX-2 in HT-29 cells was detected by Western blot and RT-qPCR. The results showed that vitexin enhanced the inhibitory effect of aspirin on COX-2 expression in HT-29 cells at the protein (**Figures 10A, B**) and mRNA (**Figure 10C**) levels after 48 h treatment, suggesting that the inhibition of colorectal cancer cell proliferation by vitexin enhanced aspirin may be achieved through the inhibition of COX-2 expression.

#### 2.4.4 Effect of aspirin-enhanced aspirin on NF-κB activity in HT-29 cells by vitexin

**Figure 11:**
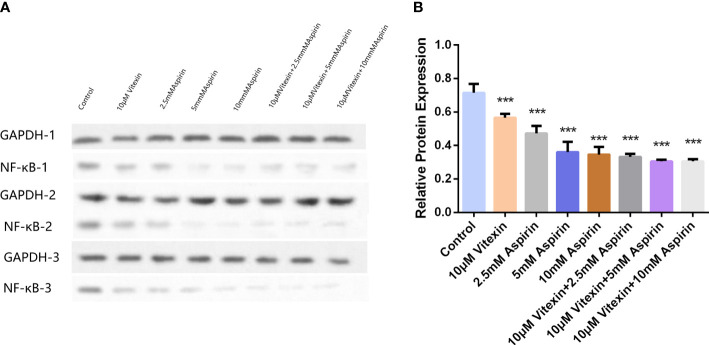
The inhibition of NF-κB expression in HT-29 cells by vitexin-enhanced aspirin was tested by Western blot. **(A)** Electrophoresis of NF-κB protein in HT-29 cells, GAPDH as loading control; **(B)** Quantifying corresponding grayscale values of Western blotting. Western blots were performed in triplicate. Each bar represents the mean ± SD from three independent assays ± SD. ***P < 0:001.

Since NF-κB can regulate the expression of COX-2, to investigate the mechanism of the anticancer activity with a focus on colorectal carcinoma of vitexin, HT-29 cells were grown in well plates, and after treating the cells in the above four ways for 48 h, and the expression of NF-κB was detected by Western blot. The results showed (**Figure 11**) that vitexin significantly enhanced the inhibition of NF-κB expression in colorectal cancer HT-29 cells by 2.5 mM aspirin, suggesting the enhanced inhibition of COX-2 expression in colorectal cancer HT-29 cells by vitexin may be achieved by inhibiting the activity of NF-κB.

## 3 Discussion

Colorectal cancer (CRC) poses a great challenge to people’s survival as a highly prevalent digestive system malignancy. In recent years, the cancer spectrum in China is changing from the developing country model to the developed country model, and the incidence rate of CRC is increasing yearly (29). According to the latest data from the National Cancer Center, colorectal cancer is currently the 3rd in the incidence spectrum of malignant tumors and the 5th in the death spectrum, which has seriously threatened the life and health of Chinese residents (30), and the incidence rate of young people has gradually increased in recent years (31). Chemotherapy is the main method used in the treatment of cancer. Unfortunately, chemotherapy destroys cancer cells, normal tissue, and cells and has significant side effects (32).

Aspirin is one of the most promising chemo preventive agents for CRC, and it has been shown that aspirin inhibits inflammation-induced colorectal carcinogenesis by reducing COX2 and ROS levels, thereby reducing DNA damage and oncogenic YAP1 (33). Studies have shown that EGFR is overexpressed in the early stage of colon cancer. Prostaglandins (PG) can upregulate the expression of EGFR in family patients with multiple adenomas, while aspirin can reduce PG synthesis by inhibiting the synthesis of COX-2 in tumor cells. Compared with family multiple adenoma patients who did not take aspirin, the expression of EGFR in adenomatous polyps was significantly decreased in patients taking aspirin (34).

Recent studies have shown that aspirin has anticancer effects on various cancers, opening up a new way to develop low-cost and low-toxic anticancer drugs (35, 36). Due to its low water solubility (pKa 3.5) and ineffective exoskeleton trapping, aspirin can retain a high concentration of circulation time in particular tumor locations. Phuong et al. established a drug delivery platform for Aspirin-loaded nano-exosomes. Using *in vivo* and *in vitro* models, it was found that these exosomes enhanced cell uptake through dependent and independent endocytosis pathways and significantly improved the cytotoxicity of aspirin to breast and colorectal cancer cells, accompanied by enhanced apoptosis and autophagy (35). To improve the effectiveness of aspirin against colorectal cancer and reduce gastrointestinal side effects, Ruberte et al. design methyl selenium-ASA analogs by doping aspirin with Se atoms and methyl selenium-ASA analogs from β- and HP-β-CDS and Pluronic F127 modified methyl selenium-ASA analogs, which exhibit a CRC cell more sustained antitumor activity (36).

Traditional Chinese medicine has unique advantages in treating colorectal cancer, which can be intervened in tumor treatment, often combined with surgical treatment, radiotherapy, and chemotherapy. Natural products are receiving increasing attention due to their advantages, such as good tolerability, lower toxicity, better therapeutic effect, and improved quality of life for cancer patients. Flavonoids have various biological activities, such as anti-inflammatory, antitumor, antioxidant, antibacterial, and antiviral. In recent years, there has been an increase in interest in the anticancer effects of flavonoids (37).

Vitexin, a natural flavonoid with a wide range of sources, has biological functions such as antioxidant and anti-inflammatory and has preventive and therapeutic effects on diseases. It also has low adverse effects and high safety compared with clinical drugs, a natural bioactive substance with great potential for development. It also has an antiproliferative effect on many tumor cells and a wide range of therapeutic effects. It exerts antitumor effects mainly by promoting apoptosis autophagy and inhibiting cell proliferation and migration of tumor cells (38). Vitexin has no significant toxic effects on normal human cells in cancer treatment and can cause apoptosis of tumor cells without damaging normal human cells (16). Several studies have indicated that vitexin can effectively inhibit the proliferation of colorectal cancer *in vivo* or *in vitro* (22–24). Therefore, it is hypothesized that vitexin is a potential anticancer chemotherapeutic agent, including colorectal cancer, and can enhance the anticancer activity with a focus on the colorectal carcinoma effect of aspirin.

To verify this hypothesis, this study explored the potential mechanisms of action of vitexin and aspirin against colorectal cancer using network pharmacology. The results suggest that vitexin and aspirin can regulate the PI3K-Akt signaling pathway, IL17 signaling pathway, TNF signaling pathway, and NF-κB signaling pathway through multiple target proteins such as NFKB1, PTGS2, MAPK1, MAPK3, and TP53, reflecting the characteristics of the new combination of vitexin and aspirin through multi-targets and multi-pathways, which echoes the complex pathogenesis of colorectal cancer. Molecular docking revealed that Aspirin-Vitexin docked with COX-2 and NFKB1 simultaneously, forming Aspirin-Vitexin-COX-2 and Aspirin-Vitexin-NFKB1 ternary complexes, both with binding affinity. The binding modes of the two ternary complexes show that the two molecules can interact well with the protein. To further elaborate on the effect of the bimolecule molecule on the protein, molecular dynamics simulations were used to show the difference in stability and binding energy of the two proteins under the action of aspirin alone and the simultaneous action of Aspirin/Vitexin. The results of molecular dynamics simulations showed that the binding of aspirin and vitexin bimolecules molecules could stabilize the conformation of NFKB1 protein and thus inhibit the protein action by synergistic action.

To further reveal the mechanism of the new combination of vitexin and aspirin against colorectal cancer, it was found that the inhibitory effect of vitexin and aspirin on the proliferation of HT-29 cells was stronger than that of the single drug group after 48 h. In addition, the results at different time points showed that the inhibition of vitexin and aspirin on cell proliferation depended on the treatment time, suggesting that the novel combination of vitexin and aspirin has some inhibitory effect on the proliferation of colorectal cancer cells.

The tumor suppressive effects of aspirin involve several molecular mechanisms, and studies have shown that aspirin targets the COX-2 enzyme and inhibits COX2-mediated production of arachidonic acid-catalyzed prostaglandins (39). At the same time, the effect of aspirin on platelets is also related to the inhibition of COX-2, which leads to the apoptosis of tumor cells and the antitumor effect by blocking lipid paracrine and COX-2 of cells adjacent to mucosal damage (40). It has been reported to be overexpressed in colorectal cancer, and the reported benefit of using aspirin to reduce the risk of colorectal cancer is almost exclusively limited to COX-2-positive colorectal cancer (41). HT-29 cells were treated with different concentrations of vitexin for 48 h. It was found that vitexin could dose-dependently inhibit the expression of COX-2 in HT-29 cells. From the level of protein and mRNA, it was found that the combination of vitexin and aspirin inhibited the expression of COX-2 in cells more obviously than in the single drug group.

Inflammation is closely associated with the development of colorectal cancer, and the exact mechanisms by which molecular markers mediate inflammatory pathways and cancer development are still under investigation. Nuclear factor-κB (NF-κB) is a well-known transcription factor that regulates gene expression in cell survival and proliferation, drug resistance, metastasis, and angiogenesis. Activation of NF-κB plays a central role in developing of inflammation and cancer (42). NF-κB is an inducible transcription factor that controls the expression of various inflammation-related genes, including COX-2. The COX-2 promoter contains two NF-κB binding sites, and NF-κB can increase COX-2 expression in response to variousf cytokines (43). It was found that activation of the NF-κB pathway may positively correlate with the degree of CRC malignancy (44). NF-κB can downstream regulate COX-2 transcription, leading to increased COX-2 expression, which promotes the production of multiple inflammatory transmitters and amplifies and sustains inflammation (45). Therefore, this study found that the combination of vitexin and aspirin inhibited the expression of NF-κB in colorectal cancer HT-29 cells more significantly than that of a single drug, suggesting that the novel combined anticancer activity with a focus on colorectal carcinoma effect of vitexin and aspirin may be achieved by inhibiting NF-κB activity and thus COX-2 expression.

## 4 Conclusion

In summary, this study used network pharmacology, molecular docking, and molecular dynamics simulations to reveal the possible mechanisms of the novel combination of vitexin and aspirin for treating of colorectal cancer. The combination of vitexin and aspirin can regulate multiple signaling pathways through target proteins such as NFKB1, PTGS2 (COX-2), MAPK1, MAPK3, and TP53. *In vitro* experiments have demonstrated that the novel combination of vitexin and aspirin has some synergistic enhancement against colorectal cancer, and the mechanism may be the inhibition of NFKB1 activity, thus inhibiting the expression of COX-2, which in turn inhibits the proliferation of colorectal cancer cells. However, our work did not allow us to determine the optimal dose of vitexin and aspirin. Therefore, more animal and cellular models are needed for future validation.

## 5 Materials and methods

### 5.1 Network pharmacology to explore the underlying mechanism of action of vitexin and aspirin against colorectal cancer

#### 5.1.1 Collection of potential targets of Vitexin and Aspirin

The molecular structures of vitexin and aspirin were obtained from the PubChem compound database (https://www.ncbi.nlm.nih.dov/) and uploaded to the Swiss Target Prediction database (http://www.swisstargetprediction.ch/), and with Probability > 0 were included in the screening to obtain the predicted genes. Meanwhile, the keywords vitexin and aspirin were used in the Encyclopedia of Traditional Chinese Medicine (ETCM) database (http://www.tcmip.cn/ETCM/index.php/Home/) and Genecards database (https://www.genecards.org/) database were searched to obtain target genes. All targets obtained by deleting duplicate target genes were selected as potential targets associated with vitexin and aspirin.

#### 5.1.2 Disease target gene collection

Using “colon cancer” and “colorectal cancer” as search terms to obtain relevant disease genes from the Gene Cards database, and filtered with the Relevance score value, the disease genes related to colorectal cancer were obtained by selecting the relevant genes with “*” sign from OMIM database. Subsequently, the drug genes and colorectal cancer targets were entered into the bioinformatic database (http://bioinformatics.cn) separately, and the intersection targets of Chinese medicine targets and disease targets were screened to construct Venn diagrams.

#### 5.1.3 Drug-disease-target network construction

Based on the target information of vitexin, aspirin, and colorectal cancer, the intersection target name, drug name, and drug composition were imported into Cytoscape3.2.1 software for visual analysis, and the network among vitexin, aspirin, colorectal cancer, and their common targets was constructed.

#### 5.1.4 Gene ontology (GO) and Kyoto encyclopedia of genes and genomes (KEGG) pathway enrichment analysis

DAVID is a bioinformatics database that integrates biological data and analysis tools to provide systematic and comprehensive bio functional annotation information for large-scale gene or protein lists to help users extract biological information (GO analysis and KEGG pathway analysis). The overlapping targets of vitexin, aspirin, and colorectal cancer were entered into the DAVID6.8 database for GO and KEGG pathway enrichment analysis. The results were plotted using the Rstudio package “ggplot2”.

#### 5.1.5 Molecular docking

The docking simulation technique is a convenient and effective for exploring small molecules’ interaction with target targets. To further investigate the mechanism of action of vitexin to enhance aspirin against colorectal cancer, we selected PTGS2 (COX-2) and NFKB1 for simultaneous molecular docking with vitexin and aspirin according to the KEGG results. The crystal structures of COX-2 (PTGS2) and NFKB1 proteins were obtained from the PDB database with PDB IDs 5IKQ and 3GUT, respectively, and the 3D structures of small molecules aspirin and vitexin were obtained from the PubChem database by downloading and energy minimization under the MMFF94 force field.

This study performed the molecular docking work using AutoDock Vina 1.1.2 software. Firstly, aspirin was docked to the already reported binding pocket of COX2 protein, while the COX2/Aspirin complex was used as the receptor, and vitexin was used as the ligand to make the docking. The pocket size was set to 90*90*90 cubic angstroms for the second docking to wrap up the possible active binding sites and finally obtain the ternary complex. For the NFKB1 protein, we performed a similar operation. Docking includes the following details, using PyMol 2.5.2 to process the receptor protein, including removing water molecules, salt ions, and small molecules. Then set the docking box. The box information is shown in **Table 1**. In addition, ADFRsuite 1.0 was used to convert all processed small molecules and receptor proteins into the PDBQT format necessary for AutoDock Vina 1.1.2 docking. In docking, the small molecule is flexible and the protein is rigid; to fully explore the binding conformation of small molecules, the detail of global search is set to 32; the algorithm used in docking is Iterated local search global optimizer. The docking conformation with the highest docking output score is considered binding conformation. Finally, PyMol2.5.2 docking ternary complex results are visually analyzed.

**Table 1:** Docking box information table.

Receptor	Ligand	Box Center	Box size
COX-2	Aspirin	37.79,35.4,24.06	25*25*25
COX-2_Aspirin	Vitexin	21.48,55.81,9.40	90*90*90
NFKB1	Aspirin	28.73,-23.68,58.06	90*90*90
NFKB1_Aspirin	Vitexin	10.72,-34.9,46.3	90*90*90

"*" represents a multiplication by "x".

#### 5.1.6 Molecular dynamics simulation (Molecule dynamics, MD)

All-atom molecular dynamics simulations were performed separately based on the small molecule and protein complexes obtained by docking as initial structures, using AMBER 18 software. Before the simulations, the charges of the small molecules were calculated using the antechamber module and Hartree-Fock (HF) SCF/6-31G* of the Gaussian 09 software. Afterward, small molecules and proteins were described using the GAFF2 small molecule force field and the ff14SB protein force field, respectively. The LEaP module was used for each system to add hydrogen atoms to the system, a truncated octahedral TIP3P solvent box was added at a distance of 10 Å from the system, and Na+/Cl- was added to the system for balancing the system charge, and finally, the topology and parameter files for the simulation were output.

Molecular dynamics simulations were performed using AMBER 18 software. Before the simulation, the system was subjected to energy optimization, including a steepest descent method with 2500 steps and a conjugate gradient method with 2500 steps. After the energy optimization of the system was completed, a 200 ps ramp-up of the system was used to slowly increase the system temperature from 0 K to 298.15 K at a fixed volume and constant ramp-up rate. A 500 ps NVT (isothermal isobaric) system simulation was performed to further uniformly distribute the solvent molecules in the solvent box at a maintenance temperature of 298.15 K. Finally, the equilibrium simulation of 500 ps was performed for the whole system under NPT (isothermal isobaric) case. Finally, 100 ns NPT (isothermal isobaric) tethered simulations were performed for each composite system under periodic boundary conditions. In the simulations, the non-bond truncation distance is set to 10 Å. The Particle mesh Ewald (PME) method is used to calculate the long-range electrostatic interaction, the SHAKE method is used to limit the bond length of hydrogen atoms, and the Langevin algorithm is used for temperature control, where the collision frequency γ is set to 2 ps-1. The system pressure is 1 atm, the integration step is 2 fs, and the trajectory is saved at 10 ps intervals for subsequent analysis, trajectories for subsequent analysis. In addition, we also used MM/GBSA with the GB8 method to calculate the binding energy of aspirin with COX-2 and NF-κB1; Aspirin with COX2-Vitexin and NF-κB1-Vitexin based on 90-100 ns simulated trajectories.

### 5.2 *In vitro* experiments

#### 5.2.1 Cell culture

The colorectal cancer cell line HT-29 cell line was purchased from Wuxi Xinrun Biological Co., Ltd (Wuxi, China). HT-29 cells were cultured in DMEM high sugar medium containing 10% fetal bovine serum, 2 mM L-glutamic acid, non-essential amino acids, and sodium pyruvate at 37°C, 5% CO_2_ in a constant temperature incubator. When the cells grow to 80-90% confluence, remove the cells from the cell culture incubator, place them in the ultra-clean bench, aspirate, and discard the medium, PBS wet wash, add 0.25% trypsin to digest for 1-2 min, add an appropriate amount of complete medium when the cells become round and not yet floating and repeatedly blow to make the cells completely and uniformly suspended, divide the cell suspension into culture dishes equally according to 1 pass 3, and place them in the incubator. The cells were incubated for 2-3 d.

#### 5.2.2 Cell viability assay

The MTT method was used to detect the growth-inhibitory effects of vitexin on different tumor cells. Four treatment methods were set up: negative control group, vitexin group, aspirin (2.5, 5, 10 mM), vitexin group, and combination group.4 groups of cells were grown in 96-well culture plates at a density of 5 ×10^3^. 10 μM vitexin was added to the vitexin monotherapy group and combination, and different concentrations (2.5, 5, and 10 mM) of aspirin were added to the aspirin monotherapy group and combination 2 h later. Three replicate wells were set up for each treatment after incubation in a constant temperature incubator at 37°C, 5% CO_2_ for 24, 48, and 72 h. The MTT storage solution at a final concentration of 0.5 mg/ml was added to each well at 10 μl per well, and after 4 h in the incubator, the medium was completely removed, and 100 μl DMSO was added to dissolve the crystals. The culture plate was placed in an enzyme marker, and the absorbance value was detected at the wavelength 570 nm to calculate the cell proliferation inhibition rate. Cell proliferation inhibition rate (%) = (average OD value of control wells - average OD value of test wells)/(average OD value of control wells - average OD value of blank wells) 100%.

#### 5.2.3 Western blot method to detect the expression of HT-29 cell-associated protein in colorectal cancer

HT-29 cells were grown in 6-well plates, treated with different drugs, and incubated for 48 h. Total cellular proteins were extracted with RIPA rapid lysate, and protein concentrations were quantified using the BCA protein analysis kit. The proteins in the cells were separated by SDS-PAGE separation gel and transferred to PVDF membranes, which were closed in 5% skim milk for 1 h, washed three times in TBST, and incubated with COX-2 (1:1000; Affinity), NF-κB monoclonal antibody (1:1000; manufacturer) monoclonal antibody. And GAPDH was used as a loading control. Then, the immunoblots were incubated with HPR-coupled goat anti-rabbit secondary antibodies (Sollerbauer, SE134) for 1 h at 37°C, stained with enhanced ECL chemiluminescent substrate, and visualized by Tanon 5200 chemiluminescent imaging system. The gray density of each protein band was normalized to the gray density of GAPDH. Each assay was repeated in triplicate.

#### 5.2.4 RT-PCR assay

HT-29 cells were grown in well plates, and the negative control group, vitexin (10 μM) drug group, aspirin (10 mM) drug group, and combination drug group were set up. HT-29 cells were treated according to these four treatments, and total cellular RNA was extracted with Trizol after 48 h incubation, and RNA samples were transcribed into cDNA, and then specific primers (**Table 2**) were used to Real-time quantitative polymerase chain reaction was performed with GAPDH as the internal control. the PCR reaction conditions were: pre-denaturation at 95°C for 5 min; denaturation at 94°C for 30 s, annealing at 60°C for 45 s, extension at 72°C for 30 s, and final extension at 72°C for 7 min, for a total of 40 cycles. The effect of different treatments on the mRNA levels of COX-2 was examined by electrophoresis on agarose gels and collecting images. Statistical analysis was performed using the 2-^ΔΔCt^ method.

**Table 2:** Primer sequence.

Name	Primer sequences
COX-2 (PTGS2)	F 5’-ATCTACGGTTTGCTGTGGGG -3’
	R 5’-TTCTGTACTGCGGGTGGAAC-3’
GAPDH	F 5’-GACAGTCAGCCGCATCTTCT-3’
	R 5’- GCGCCCAATACGACCAAATC-3’

#### 5.2.5 Statistics

Statistical analysis was performed using SPSS 21.0 software, and all test data were described as mean ± standard deviation (SD). One-way ANOVA was used for multiple group comparisons, and a t-test was used for comparing two groups. *P*<0.05 indicates that the difference is statistically significant. GraphPad Prism 6.0 software was used for image processing.

## Data availability statement

The original contributions presented in the study are included in the article/supplementary material. Further inquiries can be directed to the corresponding authors.

## Author contributions

DC: methodology, formal analysis, validation, and writing—original draft. YC: methodology, formal analysis, data curation, visualization, and conceptualization. FH: validation, investigation, and supervision. XZ: supervision and writing—review and editing. YZ and LX: resources, conceptualization, project administration, supervision, writing—review and editing. All authors have read and agreed to the published version of the manuscript.
